# Cutting edge reviews: Battling one Neglected Tropical Disease after another

**DOI:** 10.1371/journal.pntd.0014239

**Published:** 2026-04-21

**Authors:** Georgios Pappas, Paul J. Brindley, Shaden Kamhawi

**Affiliations:** 1 Institute of Continuing Medical Education of Ioannina, Loannina, Greece; 2 Department of Microbiology, Immunology and Tropical Medicine, George Washington University School of Medicine & Health Sciences, Washington,‌‌ District of Columbia, United States of America; 3 Vector Molecular Biology Section, Laboratory of Malaria and Vector Research, National Institute of Allergy and Infectious Diseases, National Institutes of Health, Bethesda, Maryland, United States of America

## Introduction

*PLOS Neglected Tropical Diseases (PLOS NTDs)* announces a commissioned reviews program led by Dr. Georgios Pappas to advance Neglected Tropical Disease (NTD) research and support 2030 elimination goals. Review editors will commission state-of-the-art, thematic, and horizon-scanning reviews selected for impact, relevance, and diverse global authorship. These high-quality syntheses aim to shape research priorities, inform practice, and serve readers as accessible resources. Prospective authors are invited to express interest and watch for forthcoming invitations and published reviews.

Dr Georgios Pappas is a physician from Ioannina, Greece, with an extended body of research on zoonoses, authoring seminal articles on human brucellosis [1–2] and epidemic preparedness and response, either natural [3], or bioterrorism-related [4–5]. As an expert on zoonoses, Dr. Pappas is also interested in social aspects of infectious diseases.

## In conversation with Georgios

### How do you see high-quality reviews shaping the future direction of the NTDs field of research?

NTDs need expansion of awareness of their burden. Kind of “in need of better public relations”. Awareness is important for securing funds to battle endemic NTDs and to ensure that, when a NTD intrudes in countries where it is traditionally absent, there is prompt recognition and a rapid response. A typical recent example would be mpox. A state-of-the-art review is an ideal introduction for the uninitiated, and of value to experts if it is up-to-date, covering all aspects comprehensively and outlining basic research or clinical gaps in our knowledge.

### In your view, what benefits or value do commissioned reviews bring to PLOS NTDs’ readership?

For the readership of PLOS NTDs (but also infectious diseases, clinical microbiology, and public health practitioners in general), such reviews will serve as the ultimate reference tool for a specific NTD. For example, it has been surprising and alarming to find that for trichinellosis and neurocysticercosis, two significant NTDs with a worldwide burden and interest, such a review does not exist in the published literature of the last decade and more. These were two of the first reviews we commissioned, and we are proud of how they turned out. Moreover, we anticipate that these reviews will allow researchers from different fields to recognize common grounds between NTDs, and thus, either apply successful practices from other diseases to their own, or understand and implement collaborative research, particularly in social aspects of NTDs.

### How will topics for commissioned reviews be selected and what criteria matter most?

The initial idea was to create a state-of-the-art, up-to-date, complete review for any individual infection or condition categorized by the journal as an NTD. An exception is NTDs for which there have been exemplary reviews published recently elsewhere, since we don’t aim to replicate but to recognize and cover gaps of knowledge. Evolving in parallel, we want to recognize situations that are developing in real time, either specifically for a disease/condition to spread the word, or for specific issues arising from the battle against NTDs—environmental or policy aspects, for example. Thus, we aspire to provide rapid, high-quality reviews of emerging situations.

### Please comment on how we will ensure a diversity of voices and global representation in our authorship of these reviews?

We actively seek renowned experts for each NTD. I begin by scanning existing and recent literature due to professional interest, but the editorial team of PLOS NTDs has been assisting me with great suggestions of topics and experts. Sometimes the experts are an up-and-coming team producing novel, interesting work, or individuals with deep knowledge of the field and exemplary ability to summarize all aspects of an NTD. NTDs are predominantly an issue of low- and middle-income countries (LMICs), so we actively work to recruit authors from endemic regions or at least build an authorship collaborative team that includes LMICs scientists. We also value equitable gender representation, selecting leaders based only on their scientific authority.

### What aspects of the reviews strategy are you personally most excited about?

The review process itself is exciting because we recruit and interact with subject experts and engage them in a constructive indirect dialogue. Reading the final version is at present the most exciting part for me, because it makes me wiser. I am looking forward to seeing these reviews getting the recognition they deserve from the scientific community, to seeing questions and novel approaches being raised “because of that review in PLOS NTDs”, and to seeing them augment the translation from interest to action where it matters most, in the field.

At the moment, four such reviews have been published or will be published in the coming days, on trichinellosis [6], neurocysticercosis [7], glanders [8], and Hendra virus [9].

At present, five additional reviews are undergoing peer review and twenty-one more have already been commissioned (thirteen on individual NTDs and eight on specialized aspects of diseases or vectors), and we are always open to external suggestions from our community. Please feel free to contact me at gpele@otenet.gr or georgiospappas70@gmail.com.

Lack of awareness is the life story of NTDs. They are diseases of the poor, of LMICs or regions, with limited opportunities for research funding. PLOS NTDs feels strongly that, as a community, we need to do more to further increase visibility of NTDs. The commissioned reviews program is one way towards accomplishing it.
